# Training with Agency-Inspired Feedback from an Instrumented Glove to Improve Functional Grasp Performance

**DOI:** 10.3390/s21041173

**Published:** 2021-02-07

**Authors:** Mingxiao Liu, Samuel Wilder, Sean Sanford, Soha Saleh, Noam Y. Harel, Raviraj Nataraj

**Affiliations:** 1 Department of Biomedical Engineering, Stevens Institute of Technology, Hoboken, NJ 07030, USA; mliu26@stevens.edu (M.L.); swilder@stevens.edu (S.W.); ssanford@stevens.edu (S.S.); 2 Movement Control Rehabilitation (MOCORE) Laboratory, Altorfer Complex, Stevens Institute of Technology, Hoboken, NJ 07030, USA; 3 Center for Mobility and Rehabilitation Engineering Research, Advanced Rehabilitation Neuroimaging Laboratory, Kessler Foundation, East Hanover, NJ 07936, USA; SSaleh@kesslerfoundation.org; 4 Spinal Cord Damage Research Center, James J. Peters VA Medical Center, Bronx, NY 10468, USA; noam.harel@mountsinai.org; 5 Departments of Neurology and Rehabilitation and Human Performance, Icahn School of Medicine at Mount Sinai, New York, NY 10029, USA

**Keywords:** hand, rehabilitation, cognition, sensory feedback, hand strength, artificial intelligence

## Abstract

Sensory feedback from wearables can be effective to learn better movement through enhanced information and engagement. Facilitating greater user cognition during movement practice is critical to accelerate gains in motor function during rehabilitation following brain or spinal cord trauma. This preliminary study presents an approach using an instrumented glove to leverage sense of agency, or perception of control, to provide training feedback for functional grasp. Seventeen able-bodied subjects underwent training and testing with a custom-built sensor glove prototype from our laboratory. The glove utilizes onboard force and flex sensors to provide inputs to an artificial neural network that predicts achievement of “secure” grasp. Onboard visual and audio feedback was provided during training with progressively shorter time delay to induce greater agency by intentional binding, or perceived compression in time between an action (grasp) and sensory consequence (feedback). After training, subjects demonstrated a significant reduction (*p* < 0.05) in movement pathlength and completion time for a functional task involving grasp-move-place of a small object. Future work will include a model-based algorithm to compute secure grasp, virtual reality immersion, and testing with clinical populations.

## 1. Introduction

Wearable sensors are becoming increasingly prevalent to monitor movement activities and to provide the user feedback, from which current and future behavior can be modified [1]. Informational updates are often provided in the aggregate (e.g., number of steps for the day), from which the user can make conscious decisions to broadly adapt future behaviors. However, sensory feedback provided with each individual motor action can continuously adapt intrinsic cognitive and behavioral patterns in performing these movements. Training with transient alerts by visual, sound, or haptic cues is proven effective to improve movement function [2] while maintaining skilled attention [3]. While sensory feedback approaches can facilitate general motor learning, they are commonly and critically evaluated for the purposes of movement rehabilitation after neurological traumas. In this context, the sensory feedback must especially ensure efficient functional progress for the patient.

Injury to the spinal cord or brain often requires physical therapy to regain functional abilities and perform activities of daily living (ADLs) [4,5]. Restoring hand control is especially critical for interacting with the environment and improving quality of life [6]. Traditional physical therapy can be intensive and repetitive; however, it can be difficult to maintain the continued participation that is necessary to regain neuromotor function [7]. Novel rehabilitation methods such as virtual reality (VR) [8] are increasingly employed to facilitate greater motivation and cognitive engagement through stimulating feedback while performing functional tasks. Engagement during physical therapy after neurotraumas is challenging but critically important, and rehabilitative devices that further leverage cognition may be the key for continued and efficient progress.

Instrumented gloves for rehabilitation commonly provide physical assistance or guidance to the user through robotic-type control and actuation [9,10]. Alternatively, sensor gloves [11,12] collect information about hand movements from which sensory cues can be provided. We have developed an instrumented glove system that does not *physically* assist, but rather provides informative feedback about secure grasp to *cognitively* engage the user during training. Secure grasp can be analytically identified by deterministic factors of grasp force quality [13]. Secure grasp may also be detected empirically via machine learning algorithms that discern functionally effective grasp patterns [14]. Methods that effectively inform about functional performance while shaping perception may unlock greater potential in grasp learning applications, including rehabilitation.

Sense of *agency* is the perception of control over one’s own actions [15]. Because agency and movement performance are positively related [16], we posit that rehabilitation training methods that leverage cognitive agency may accelerate positive functional outcomes. To this end, we have developed an instrumented glove that includes onboard sensors, computational capabilities, and sensory performance feedback modules capable of the following objectives: (1) identifying achievement of secure grasp; (2) informing the user with multimodal feedback; (3) manipulating feedback timing to strengthen “intentional binding”. Intentional binding is the compression of the user’s perception of time between voluntary action and expected consequence, and it serves as an implicit measure of agency [17]. To express greater agency during feedback training, one would further perceptually link movement actions to consequences such as sensory cues, which directly result from those actions.

In this pilot investigation, we employed cognitive agency-based training with our sensor glove through gradual reduction in the time-interval between action (grasp) and consequence (sensory feedback). By progressively shortening the delay in sensory feedback, we expect perceptual conditioning that facilitates agency-based performance gains. As proof-of-principle, we utilized machine learning (artificial neural network) to detect secure grasp from force- and flex-sensors on the glove and to subsequently trigger multimodal (visual, audio) sensory feedback during a block of training trials. We hypothesized that training with agency-based feedback will further improve performance of a functional grasp task compared to no feedback or immediate feedback. This preliminary work with healthy persons will serve as an important first step to characterize functional motor responses when feedback from a sensor glove is provided at timing intervals intended to condition intentional binding, a well-accepted surrogate for agency. These findings for a singular principle may motivate consideration of how cognitive variables could be better leveraged with modified presentations of sensory feedback for more effective motor learning. Cognition-based motor principles established at a fundamental level with healthy persons using this sensor glove can stimulate pursuit of cognitive-centered design of motor rehabilitation paradigms. In addressing various clinical populations with diverse neuromotor disease phenotypes, future work may then build upon such principles with more sophisticated and customized approaches involving virtual reality with instrumented wearables.

## 2. Materials and Methods

This experimental design of this study involved testing of healthy subjects performing functional grasp-lift-place of a small object after receiving sensory feedback training at specific time-intervals in relation to achieving secure grasp. As detailed below, there were three training groups whereby feedback was: (1) not received, (2) received immediately, or, (3) received at progressively shorter time-intervals. This third group served as the one intended to condition agency via intentional binding. Variability in the experimental design was primarily minimized three ways: (1) use of the same subjects across all three feedback modes, (2) use of the same glove and feedback cues, and (3) a functional task commonly employed for clinical grasp rehabilitation [18,19,20]. Our subsequent data analysis was based on this 1-way design (single factor of feedback group) to evaluate three performance metrics (timing, movement pathlength, and placement accuracy), all of which are highly coupled to the task specifications. As such, any variability observed was largely attributed to effects from sensory feedback training. Since feedback was provided in accordance to training neural network output based on sensor inputs, we also include evaluations for network training and general prediction capabilities.

### 2.1. Subjects

Seventeen able-bodied subjects (11 males, 6 females, aged 23 ± 3 years) participated in this study with procedures approved by the Stevens Institutional Review Board (IRB, protocol 2017-023, originally approved May 2017 and then annually renewed). All recruited participants signed an IRB-approved informed consent form in accordance with [21]. Experimental procedures were explained and executed consistent with guidelines from [22] intended to ensure subject well-being and data credibility, among other good practices for human subject research. *Inclusion criteria*: All subjects were right-handed and should not report nor indicate complications involving cognition or upper extremity function as specified for persons with hemiparesis [23].

### 2.2. Equipment for Operating the Instrumented Glove

**Figure 1:**
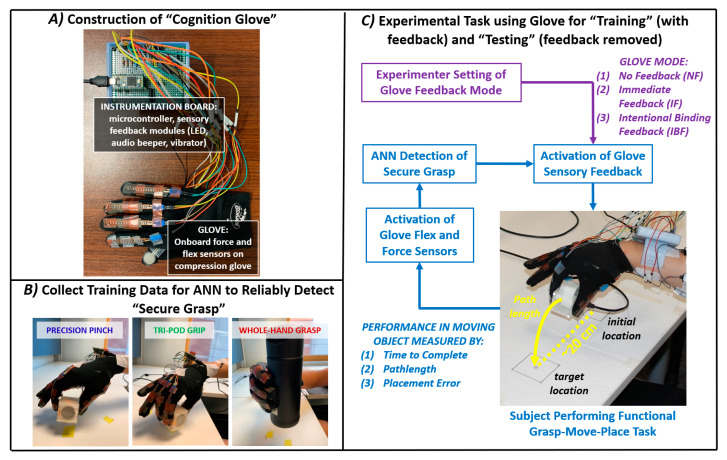
(**A**) Cognition glove hardware. Physical components, including instrumentation, of glove worn by user, (**B**) Training. Data collected to train artificial neural network (ANN) to identify secure grasp onto object across three grip types, (**C**) Functional task. Flow diagram of experiment to verify improvement in performance of grasp-move-place task when receiving feedback from glove if secure grasp achieved.

The glove hardware (Figure 1A) included a compression glove embedded with force (Interlink Electronics, Camarillo, CA, USA) and flex (Spectra Symbol, Salt Lake City, UT, USA) sensors across each digit. The flex sensors were thin and aligned only on the dorsal side, which minimized perceived changes in hand dexterity. The sensors were connected to an instrumentation board (Teensy, SparkFun, Boulder, CO, USA) programmed with Arduino (Somerville, MA, USA). The board and wired connections were housed in a 3D-printed enclosure with strap-mount to the wrist. Sensory modules included an LED (Lite-On) and an audio beeper (TDK) to provide visual and sound feedback. Vibration motors (Adafruit) for tactile feedback were available but not utilized in this study. The glove with onboard instrumentation has mass under 100 g. API code in MATLAB^®^ (Mathworks, Natick, MA, USA) read sensor data via serial communication at 40 Hz and was processed on an Intel desktop computer (Xeon^®^ 3.20GHz, 32 GB Ram, Windows 10 Pro).

### 2.3. Additional Equipment for Running Functional Tasks

A 3D-printed cubic (4 cm/side) object was used for a precision pinch (index finger and thumb) functional task (described in Section 2.5. The object was instrumented with force sensors (Ohmite, Warrenville, IL, USA) to validate glove force measurements (results not reported in this study). A marker-based motion capture system was used to track 3D position of the object during the functional task. The system included nine infra-red cameras (*Prime 17W*, Optitrack^®^, NaturalPoint, Corvalis, OR, USA) recording at 120 Hz.

### 2.4. Experiment Protocol to Train Glove on Secure Grasp

Training data was collected for each subject session involving trial-repetitions of grasp with various grips while wearing the glove (Figure 1B). For precision pinch and tri-pod grip (thumb, index, middle digits), the cubic object was grasped. For whole-hand grasp, a cylindrical container was grasped. Each repetition was ten seconds. The subject was first cued to move the gloved hand from an initial resting position (palmar side down) on the table. The subject grasped and lifted the target object approximately 2 cm off the table and would securely “hold” steady for five seconds, as cued by the experimenter. Finally, the subject re-placed the object on the table and returned the hand to rest to complete the trial. Subjects completed twenty trial-repetitions for each grip. Another twenty trials were similarly performed with each grip except the subject would continuously “tap” (grasp and immediate release) the object rather than hold in place. These tapping trials provided additional training examples for insecure grasp contact. Five other trials were performed where the gloved hand remained entirely at “rest”.

Sensor data from these trials served to subsequently train an artificial neural network (ANN) for each subject. The ANN (feedforward, two layers, ten hidden-layer neurons) was trained to discriminate “secure” and “insecure” grasp. Time instances within trials where sensor voltages were within ± 10% of the mean values during the steady “hold” period, as identified by experimenter, were classified as “secure” grasp with output value of ‘1’. All other trial data were classified as “insecure” grasp with output value of ‘0’. The scaled conjugated gradient backpropagation algorithm was used for training with 70% of the data. The training objective function was cross entropy between network and actual (target) outputs. The remaining data were distributed evenly for testing (15%) and validation (15%). The trained ANN could produce continuous output over the interval [0,1]. During the functional task, trained ANN output was rounded. ANN output values greater than 0.5 were rounded to ‘1’ to indicate secure grasp, and output values below 0.5 were rounded down to ‘0’ to indicate insecure grasp.

### 2.5. Experiment Protocol for Functional Task

Each subject participated in three separate sessions for performing a functional grasp-move-place task with the glove (Figure 1C). Each session was separated by at least three hours. The task required three steps: (1) reaching to execute precision pinch grasp onto the cubic object, (2) lifting and moving the object from its initial location, and, (3) accurately placing the object onto its designated target location (~20 cm to the left, squared outline matching object). Subjects were instructed to minimize the motion pathlength of the object, to accurately place the object onto the designated target, and to complete the task at a timely pace. For each session, the subject would perform a “baseline” block of 15 trials with no glove feedback. Following a rest period (2–3 min), the subject performed a “training” block of 30 trials with one mode of glove feedback (see below). Following another rest period, the subject performed a “post-training retention” block of 15 trials with no glove feedback. For the training block, feedback was provided as a singular audio beep (moderate tone and pitch, 100 msec duration) to alert the user that secure grasp was achieved according to output from the subject-specific ANN. Feedback was additionally provided by an LED light activating simultaneously to the audio beep and remaining activated until ANN output of secure grasp concluded due to object release. Each training block employed one of the three feedback modes: (1) “no feedback” (NF) to serve as the control group, (2) “immediate feedback” (IF) upon achieving secure grasp, or (3) “intentional binding feedback” (IBF) with time-interval delay that progressively reduced from 1 to 0 s over the 30 trials at a fixed-interval (~34 msec) per trial.

### 2.6. Data and Statistical Analysis

Improvement in performance of the functional task was measured as reduction in the following three metrics from baseline to post-training retention: (1) task completion time, (2) motion pathlength of object, and (3) placement error. Motion pathlength was computed according to accumulation of 3D position displacements of object over time samples (100 Hz) when velocity was non-zero. The completion time was measured as the time duration when object velocity was non-zero. Placement error was computed as the projection of the center of the object onto the table surface from the center of target. Procedures for marker-based digitization [24] was used to compute these center positions from reference marker-clusters located on the object and table. Prior to statistical analysis, the mean pre-training value of each performance metric for each subject was utilized to normalize training and post-training performance values for the same subject. Intra-subject normalization was used to remove potential data skewing due to possibly high inter-subject variability. To present data in real-world units, performance values were then de-normalized by multiplying the mean pre-training value for the subject group (all 17 subjects).

For these performance metrics, two training outcomes measures were specifically evaluated. First, an intra-training rate was evaluated as the fitted linear regression slope to the performance metric data plotted across thirty sequential trials during the training trial-block. Since the desired behavior is a reduction in each performance metric, a negative slope served as a ‘positive’ intra-training rate. Ultimately, we observed three distinct subject-cases whereby a given subject may exhibit a distinct combination of intra-training rates across the three training feedback modes. The second outcomes measure of interest was post-training effect whereby the difference in performance metric from pre-training to post-training trial blocks are evaluated. Again, a negative difference, i.e., reduction in performance metric value, is the desirable outcome.

For evaluating ANN training parameters, an ANOVA and Tukey post hoc comparisons with Bonferroni correction were performed for cross-entropy and percentage error across data sets for training, validation, and testing. A two-sample t-test was used to compare mean squared error computed directly from continuous ANN output versus when ANN output was rounded to 0 or 1. One-way repeated-measures ANOVA and Tukey post hoc comparisons with Bonferroni correction were performed independently for each metric across the three feedback modes serving as training groups and across trial-block times (pre-training, training, post-training). Another one-way ANOVA was done on the outcome measure of intra-training rate and post-training effect for each performance metric for comparison between training feedback groups. A one-sample t-test was also done on both outcome measures of each performance metric and feedback group to verify significant difference from zero.

## 3. Results

### 3.1. ANN Training Results

**Figure 2:**
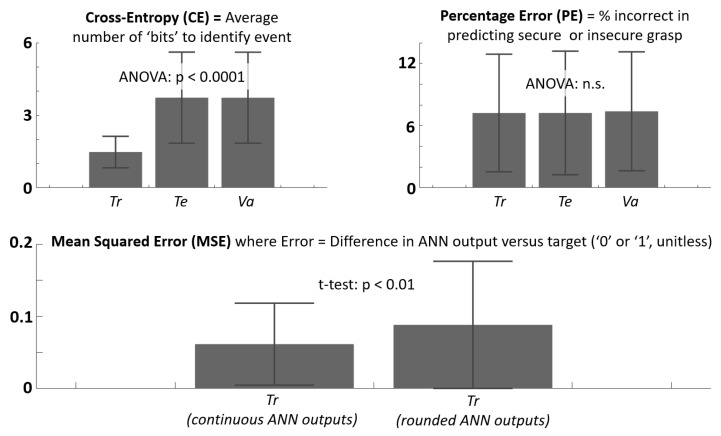
Mean values shown for artificial neural network (ANN) training parameters (CE, PE, two versions of MSE). Results for CE and PE shown for training (Tr), testing (Te), and validation (Va) data sets. Minimization of CE was objective function during training. Computation for MSE during training shown for continuous ANN output (any value over [0, 1]) versus rounded ANN output (ANN output converted to either ‘0’ or ‘1’ if continuous output is > or <0.5, respectively). Note: *p*-value > 0.05 is not significant (n.s.).

The results for ANN training parameter values are shown in Figure 2 and Table 1. The objective function was significantly minimized for the training data set relative to the validation and testing data sets as designed. However, the percentage prediction errors were similar across all three data sets, suggesting that the early-stop criterion to prevent overfitting to the training data set was desirably enacted. Two cases of mean squared error (MSE) are shown, the first (continuous) indicates errors between true ANN output versus discretized target values (0 or 1), while the second indicates average errors when ANN output is rounded to 0 or 1, as to be done during the functional task. Rounded MSE was significantly greater, but MSE for each case was desirably low (mean < 0.1).

### 3.2. Machine-Learning Detection of Secure Grasp

**Figure 3:**
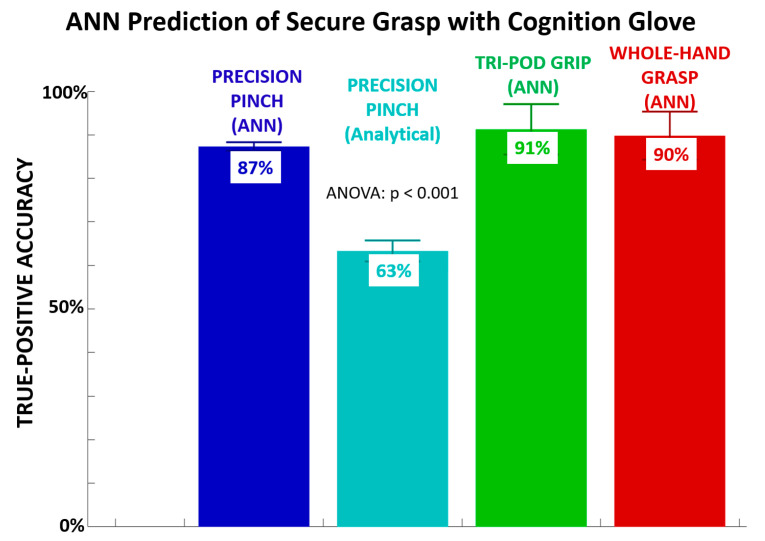
True positive rate detection of secure grasp depended if ANN utilized for prediction of precision pinch, tri-pod grip, or whole-hand grip versus analytical (cancellation) method utilized for precision pinch.

Across all subjects, the mean true-positive rate for ANN prediction of secure grasp on the testing data ranged from 87% to 91% for the three grip postures (Figure 3, Table 2). ANN prediction outperformed an analytical method for precision pinch. The analytical method was based on simple grasp force equilibrium where index and thumb force sensor voltages were sufficiently equal (within 10% of each other) and sufficiently non-zero (>10% maximum output). Voltage-based analytical methods for tri-pod and whole-hand grips are omitted as simple voltage cancelation with more than one opposing digit sensor to the thumb was not feasible. For these grips recruiting more digits, there was less opposition of the thumb pad on the object surface to generate sufficiently large readings on the thumb sensor for analytical cancelation, whereby prediction rates were poor (<50%). The true-positive rate for each grip with ANN prediction was significantly greater than the analytical method examined for precision pinch.

### 3.3. Glove Feedback Effects on Grasp Performance

**Figure 4:**
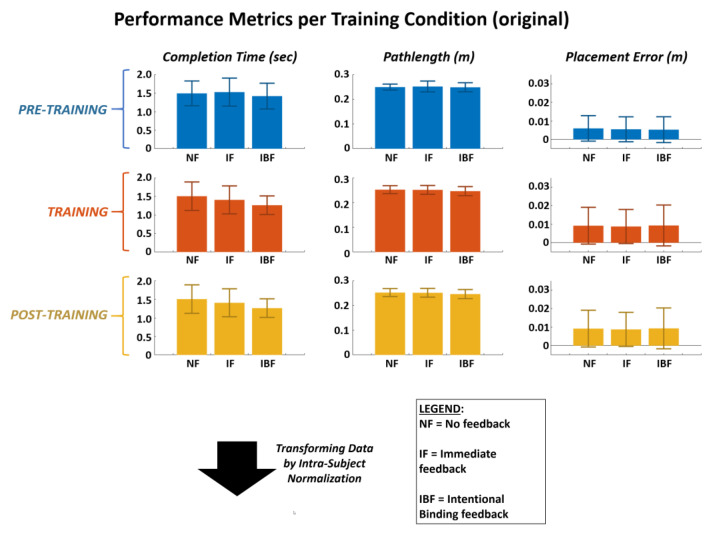

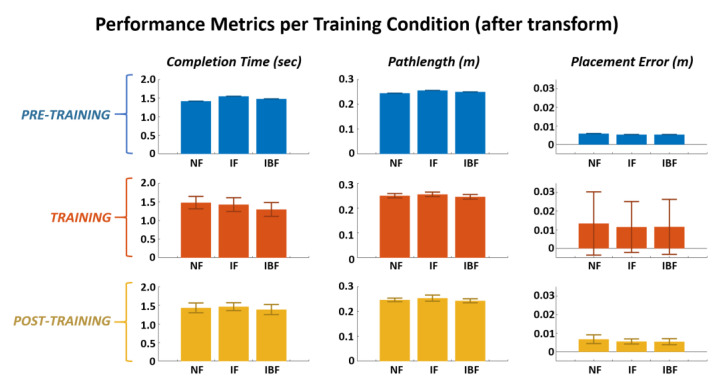
Mean values shown for performance metrics (*completion time, pathlength, and placement error*) across trial-blocks in time (*pre-training, training, post-training*). Data shown as original raw values (TOP) and after transformation (BOTTOM). For data transformation, mean pre-training value for each subject utilized to normalize training and post-training values for same subject. Metric values then de-normalized by multiplying the mean pre-training value for entire subject group.

The completion time, pathlength, and placement error data before and after transformation by intra-subject normalization from pre-training data are shown in Figure 4. The main effect, qualitatively observed, on the training and post-training performance data is reduction in the standard deviations except for placement error during training. This general reduction in standard deviation indicates presence of notable inter-subject variability that needed to be considered when evaluating effects of the glove for each person. As such, it was necessary to normalize performance data within each subject prior to pooling results across the entire subject group.

The mean pathlength, completion time, and placement error prior to training were 25.0 ± 1.75 cm, 1.48 ± 0. 35 s, 5.6 ± 0.68 mm, respectively, over all subject pre-training trial-blocks. Repeated measures ANOVA (Table 3) demonstrated significant performance differences from pre-training to either training or post-training blocks only for two metrics (completion time, motion pathlength) and training feedback modes of immediate feedback (IF) or intentional binding feedback (IBF). The IBF group signified the one having elicited greater agency through conditioning of intentional binding. These significant differences were always desirable, i.e., reduction in metric from pre-training value. Both IF and IBF generated significant differences for completion time, but only IBF did so for pathlength. The no feedback (NF) mode did not result in improvement for any metric.

**Figure 5:**
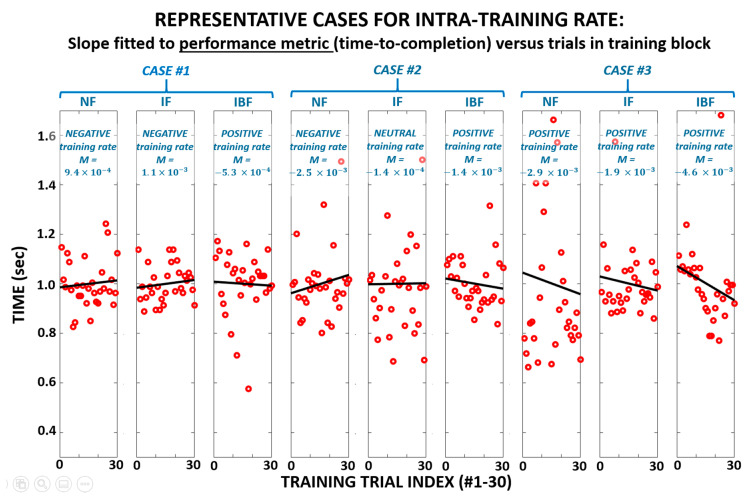
Example of linear regression fits to performance data of a single metric (completion time) shown across thirty sequential training trials for each of three feedback training conditions. Each ‘case’ represents the same subject with a distinct combination in trends of the regression slopes, i.e., intra-training rates. A ‘positive’ (desirable) intra-training rate is a decline in the performance metric value, i.e., slope < 0. *Note:* Training feedback groups: NF = No Feedback, IF = Immediate Feedback, IBF = Intentional Binding Feedback

**Figure 6:**
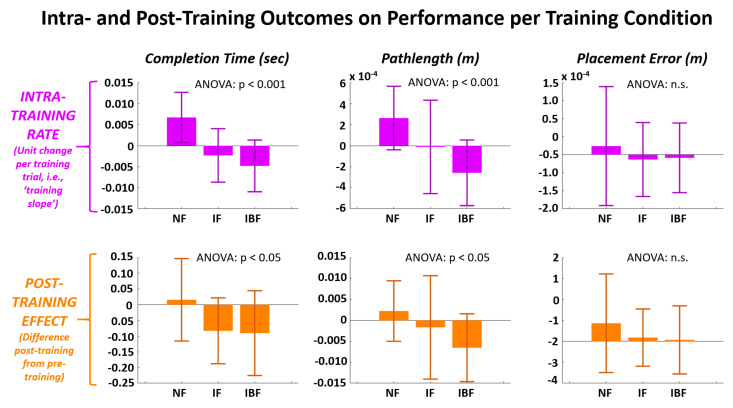
Intra-training rate and post-training change shown for each performance metric and compared across training feedback groups (NF = No Feedback, IF = Immediate Feedback, IBF = Intentional Binding Feedback). Intra-training rate indicates the average change in performance metric per training trial. Post-training change is difference in performance metric during post-training trial-block compared to pre-training trial-block.

Figure 5 shows examples for three distinct subject-cases whereby a given subject may exhibit a unique combination of intra-training rates across the three training feedback modes. Figure 6 and Table 4 show results for the two outcome measures for intra-training and post-training effects. Significant differences (*p* < 0.05) were observed across feedback groups for both completion time and pathlength for both intra-training rate and post-training effect. While IF only demonstrated significant improvement over NF for intra-training completion time, IBF demonstrated significant improvement over NF for intra-training completion time, intra-training pathlength, post-training completion time, and post-training pathlength. No significant differences were observed related to placement error.

While the ANOVA results on outcome measures indicate significant differences among feedback groups, Table 5 demonstrates whether the outcome measures were significantly non-zero. Only IBF demonstrated significant improvements in performance (i.e., reductions in metric values) from zero, and did so for intra-training completion time, intra-training pathlength, post-training completion time, and post-training pathlength. It is notable that NF did demonstrate a significant decrease in performance (i.e., increase in metric value) from zero for intra-training rate for completion time and pathlength. This result for NF demonstrated the need to show significant differences for IF and IBF not only against NF, but also a zero-reference.

## 4. Discussion

This study observed improvement in grasp performance metrics after sensory feedback training with an instrumented glove designed to induce greater cognitive agency through intentional binding. The glove hardware components are not wholly unique to previous sensor gloves. Sensor integration with a computational framework to assess secure grasp and alert the user at cognitively inspired timing intervals is the novel feature. As such, this study demonstrates an important bridging concept between the psychology of agency and delivery of sensor-based sensory feedback for improving movement performance. Specifically, post-training functional performance may be enhanced when robust computational detection of secure grasp from sensor signals is used to inform the user at specific timing intervals. The instrumented glove system was developed to alert the user when secure grasp onto an object was achieved. Training a feedforward artificial neural network on empirical data to predict all-or-nothing accomplishment of secure grasp was sufficient to examine the effects of feedback timing on performance. ANN training parameters were stable (low cross entropy, low percent error, low MSE) across training/testing/validation data sets and behaved functionally well (high true-positive prediction rate) across various grasp types. We confirmed the ANN (or equivalent computational intelligence) was needed to effectively identify empirical patterns of secure grasp. An analytical computation of secure grasp relying on explicit sensor signals would have been relatively inferior in prediction of secure grasp during training.

With ANN-based feedback, significant (*p* < 0.05) improvements in performance were observed for metrics of pathlength and completion time with intentional binding feedback, whereby feedback was provided at progressively shorter time intervals during training. Significant differences were not observed for placement error, suggesting little margin of placement variability for healthy subjects. A placement accuracy objective was still necessary to ensure subjects properly performed the task. Previous studies have demonstrated that feedback to reinforce performance and activate sensory modalities (vision, touch, hearing) can enhance movement learning [25].

Results from our study suggest that well-placed timing of sensory feedback can further accelerate functional gains. During training with IBF, we employed progressive reduction in the time-delay between voluntary grasp action and the sensory-based alert to promote greater agency by altering perception between grasp action and expectation of sensory consequences during training. Provision of feedback in this manner produced significant improvements in performance criteria of task completion time and motion pathlength compared to the control case of NF. While IF also demonstrated improved performance compared to NF, the instances were fewer and non-unique relative to IBF. Training with IBF not only produced greater significant improvements in performance measures from NF, but these improvements were all significantly non-zero. This distinction was important since NF produced instances of significantly worse post-training performance. Performance decline with NF may be attributable to cognitive fatigue from lack of engagement with repetition of a simple task whereby provision of any feedback could generate positive training and post-training effects. We postulate that larger improvements with this type of feedback training may be observed where ranges in performance are expectedly larger. This may be the case with clinical populations, complex tasks, or more enhanced feedback as with VR [8]. We further contend that coordinating sensor-based feedback to exercise the person’s perception of time delay between their voluntary actions and sensory consequences may be key to maximize positive outcomes.

Training with gradual reduction in feedback delay was inspired by the concept of intentional binding. We sought to actively compress the perceived time-interval between grasp action and the expected sensory consequence [17] as a vehicle to enhance training effects. Sensory feedback has demonstrable effects in modulating agency [15] and training [25] of movement. As such, sensory feedback approaches could be designed to leverage agency for rehabilitation training. Our study indicates that gradual reduction in the time-interval between a successful movement action and sensory feedback cues during training may condition perceptions to achieve agency-based gains in functional performance. These findings should motivate the design of sensor-based rehabilitation protocols and interfaces that specifically consider cognitive factors such as agency. Optimal user control of powered assistive devices, such as prostheses and exoskeletons, may also be better achieved with cognitive-based approaches.

The notable limitation in this study was that we did not explicitly measure agency and directly relate it to performance. Our previous work has made the positive connection between agency and performance [16] but relied on verbal estimation of randomly presented time-intervals [15] to assess agency. In this study, subject onus to estimate time-intervals may be prohibitive to the perceptual conditioning we sought to achieve. Furthermore, time-intervals were non-random (either no delay or progressively reduced delay), making implicit inferences of agency potentially irrelevant if these patterns were recognized. For our study, trial-to-trial validation of agency may require physiological indicators such as brain activity [26]. Another study limitation was that subjects informally reported they perceived audio feedback as the dominant sensory modality. The visual cue with LED was cited as relatively too subtle given the visual attention paid to performing the task. As such, enhanced visual modalities such as virtual reality may be necessary to stimulate effects beyond the audio cues alone. The LED cue may be practically useful when audio feedback is otherwise untenable, such as when performing ADLs in shared community environments. The final limitation in this study was that the subject group included only healthy participants. Ultimately, we will utilize this glove system with persons undergoing physical therapy to recover motor function after neurotraumas such as brain and spinal cord injury. As such, vibration feedback from the glove was not employed in this study since all subjects were able-bodied and did not benefit from tactile enhancement. We expect vibration cues may be effective with clinical populations having tactile deficits [27].

In the future, enhanced forms of rehabilitation should employ instrumented wearables that are easy to don-and-doff and approaches that cognitively engage participants. Our pilot study suggests that cognitive factors may be directly leveraged for better movement training through sensory feedback. Wearables that are lightweight and cosmetic could be discreetly utilized to provide cognitively inspired feedback to train better movement throughout the day. Furthermore, instrumented wearables, such as the presented glove, could facilitate usage at home and throughout the day to bridge and supplement traditional physical therapy sessions. Physical therapy itself may benefit from employing instrumented wearables and other methods that focally consider cognitive factors such as agency. We plan to make this glove compatible with VR such that it could be used in stand-alone mode while doing ADLs or incorporated with VR for intensive physical therapy. Both instrumented wearables and VR applications offer unique advantages of logging data for long-term assessment of progress, enhanced movement tracking with high-fidelity sensors or computation, and entertaining interfaces to inform and engage users. Sophisticated algorithms may be employed to further advance these features of rehabilitation systems. These algorithms can analytically identify movement features such as grasp or intelligently present feedback cues through devices and VR to personalize and optimize rehabilitation outcomes.

## Figures and Tables

**Table 1:** Mean value comparisons for ANN training parameters.

	ANN Data Set			ANOVA		Tukey post hoc		
METRIC	Training (Tr)	Validation (Va)	Testing (Te)	*p*-value	F-stat	Tr vs Va	Tr vs Te	Va vs Te
Cross-Entropy	1.48 ± 0.65	3.73 ± 1.89	3.73 ± 1.88	**8.7 × 10^−5^**	11.4	**4.0 × 10^−4^**	**4.0 × 10^−4^**	1
Error (%)	7.22 ± 5.67	7.22 ± 5.96	7.38 ± 5.73	0.99	0.0042	1	0.99	0.99
**METRIC**	**Standard MSE**		**Rounded MSE**		**T-test *p*-value**		**T-statistic**	
Mean Squared Error (MSE, unitless)	0.062 ± 0.057		0.083 ± 0.088		**3.6 × 10^−3^**		3.41	

Note: significant (<0.05) *p*-values bolded.

**Table 2:** Mean value comparisons for prediction (true-positive rate, %) of secure grasp.

Pinch ANN (1)	Pinch Analytical (2)	Tri-pod ANN (3)	Whole-Hand ANN (4)	ANOVA F-stat	ANOVA *p*-value
87.3 ± 1.0%	63.3 ± 2.4%	91.3 ± 5.8%	89.8 ± 5.5%	29.4	**1.1 × 10^−4^**
**Post hoc** **1 vs 2**	**Post hoc** **1 vs 3**	**Post hoc** **1 vs 4**	**Post hoc** **2 vs 3**	**Post hoc** **2 vs 4**	**Post hoc** **3 vs 4**
**5.3 × 10^−4^**	0.67	0.88	**1.8 × 10^−4^**	**2.6 × 10^−4^**	0.97

Note: significant (<0.05) *p*-values bolded.

**Table 3:** Repeated measures ANOVA across trial-block times (pre-training, training, post-training) for each performance metric and training condition (feedback mode); Trial-Block Time Indices: 1 = Pre-training, 2 = Training, 3 = Post-training; Feedback Modes: NF = No Feedback, IF = Immediate Feedback, IBF = Intentional Binding Feedback; ‘Difference’ is computed from performance metric value for Time 1 – Time 2.

**Performance metric: COMPLETION TIME (sec)** **→ ANOVA *p*-value = 4.9× 10^−4^, F-stat = 5.51**					
**Feedback Mode**	**Time 1**	**Time 2**	**Difference**	**Std Err**	**Post hoc *p*-value**
NF	1	2	−0.063	0.043	0.32
NF	1	3	−0.016	0.030	0.86
NF	2	3	0.048	0.040	0.47
IF	1	2	0.12	0.043	**0.021**
IF	1	3	0.083	0.030	**0.022**
IF	2	3	−0.037	0.040	0.63
IBF	1	2	0.18	0.043	**0.00039**
IBF	1	3	0.09	0.030	**0.012**
IBF	2	3	−0.09	0.040	0.077
**Performance metric: PATHLENGTH (m)** **→ ANOVA *p*-value = 0.019, F-stat = 3.10**					
**Feedback Mode**	**Time 1**	**Time 2**	**Difference**	**Std Err**	**Post hoc *p*-value**
NF	1	2	−0.0052	0.0022	0.057
NF	1	3	−0.0023	0.0023	0.59
NF	2	3	0.0029	0.0020	0.31
IF	1	2	0.00032	0.0022	0.98
IF	1	3	0.0017	0.0023	0.75
IF	2	3	0.0013	0.0020	0.78
IBF	1	2	0.0045	0.0022	0.11
IBF	1	3	0.0065	0.0023	**0.018**
IBF	2	3	0.0020	0.0020	0.57
**Performance metric: PLACEMENT ERROR (m)** **→ ANOVA *p*-value = 0.99, F-stat = 0.033**					
**Feedback Mode**	**Time 1**	**Time 2**	**Difference**	**Std Err**	**Post hoc *p*-value**
NF	1	2	−0.0075	0.0037	N/A
NF	1	3	−0.00086	0.00044	N/A
NF	2	3	0.0066	0.0037	N/A
IF	1	2	−0.0061	0.0037	N/A
IF	1	3	−0.00018	0.00044	N/A
IF	2	3	0.0059	0.0037	N/A
IBF	1	2	−0.0062	0.0037	N/A
IBF	1	3	−0.00007	0.00044	N/A
IBF	2	3	0.0061	0.0037	N/A

Note: significant (<0.05) *p*-values bolded.

**Table 4:** ANOVA for intra- and post-training performance outcomes between training conditions (feedback modes). Feedback Modes: NF = No Feedback, IF = Immediate Feedback, IBF = Intentional Binding Feedback.

**Intra-Training Rate/Slope for COMPLETION TIME (sec per trial)**							
Feedback Mode			ANOVA		Tukey post hoc		
NF	IF	IBF	*p*-value	F-stat	NF vs IF	NF vs IBF	IF vs IBF
0.0067 ± 0.006	−0.0023 ± 0.0064	−0.0048 ± 0.0061	**3.6 × 10^−6^**	16.5	**2.6 × 10^−4^**	**5.0 × 10^−6^**	0.47
**Intra-Training Rate/Slope for PATHLENGTH (m per trial)**							
Feedback Mode			ANOVA		Tukey post hoc		
NF	IF	IBF	*p*-value	F-stat	NF vs IF	NF vs IBF	IF vs IBF
2.64 × 10^−4^ ± 3.02 × 10^−4^	−1.15 × 10^−4^ ± 4.44 × 10^−4^	−2.58 × 10^−4^ ± 3.12 × 10^−4^	**4.84 × 10^−4^**	8.9	0.076	**3 × 10^−4^**	0.12
**Intra-Training Rate/Slope for PLACEMENT ERROR (m per trial)**							
Feedback Mode			ANOVA		Tukey post hoc		
NF	IF	IBF	*p*-value	F-stat	NF vs IF	NF vs IBF	IF vs IBF
2.40 × 10^−5^ ± 1.65 × 10^−4^	−1.32 × 10^−5^ ± 1.03 × 10^−4^	−8.63 × 10^−6^ ± 9.67 × 10^−5^	0.6434	0.45	N/A	N/A	N/A
**Post-Training Effect (Difference After Training from Before) for COMPLETION TIME (sec)**							
Feedback Mode			ANOVA		Tukey post hoc		
NF	IF	IBF	*p*-value	F-stat	NF vs IF	NF vs IBF	IF vs IBF
0.0156 ± 0.1312	−0.0831 ± 0.105	−0.090 ± 0.135	**0.028**	3.8	0.064	**0.043**	0.98
**Post-Training Effect (Difference After Training from Before) for PATHLENGTH (m)**							
Feedback Mode			ANOVA		Tukey post hoc		
NF	IF	IBF	*p*-value	F-stat	NF vs IF	NF vs IBF	IF vs IBF
0.0022 ± 0.0072	−0.0017 ± 0.0123	−0.0065 ± 0.0081	**0.034**	3.6	**0.46**	**0.026**	0.30
**Post-Training Effect (Difference After Training from Before) for PLACEMENT ERROR (m)**							
Feedback Mode			ANOVA		Tukey post hoc		
NF	IF	IBF	*p*-value	F-stat	NF vs IF	NF vs IBF	IF vs IBF
8.65 × 10^−4^ ± 0.0024	1.81 × 10^−4^ ± 0.0014	7.06 × 10^−5^ ± 0.0016	0.40	0.94	N/A	N/A	N/A

Note: significant (<0.05) *p*-values bolded.

**Table 5:** One-sample t-test results to confirm if intra- and post-training outcomes on performance metrics are non-zero. Feedback Modes: NF = No Feedback, IF = Immediate Feedback, IBF = Intentional Binding Feedback.

**Intra-Training Rate/Slope for COMPLETION TIME (sec)**					
NF		IF		IBF	
*p*-value	T-stat	*p*-value	T-stat	*p*-value	T-stat
**0.0003**	4.65	0.15	−1.50	**0.0053**	−3.22
**Intra-Training Rate/Slope for PATHLENGTH (m per trial)**					
NF		IF		IBF	
*p*-value	T-stat	*p*-value	T-stat	*p*-value	T-stat
**0.0024**	3.59	0.92	−0.10	**0.0036**	−3.41
**Intra-Training Rate/Slope for PLACEMENT ERROR (m per trial)**					
NF		IF		IBF	
*p*-value	T-stat	*p*-value	T-stat	*p*-value	T-stat
0.56	0.60	0.60	−0.53	0.72	−0.368
**Post-Training Effect (Difference After Training from Before) for COMPLETION TIME (sec)**					
NF		IF		IBF	
*p*-value	T-stat	*p*-value	T-stat	*p*-value	T-stat
0.63	0.49	**0.0049**	−3.27	**0.0139**	−2.76
**Post-Training Effect (Difference After Training from Before) for PATHLENGTH (m)**					
NF		IF		IBF	
*p*-value	T-stat	*p*-value	T-stat	*p*-value	T-stat
0.22	1.28	0.59	−0.55	**0.0044**	−3.32
**Post-Training Effect (Difference After Training from Before) for PLACEMENT ERROR (m)**					
NF		IF		IBF	
*p*-value	T-stat	*p*-value	T-stat	*p*-value	T-stat
0.15	1.51	0.59	0.55	0.86	0.18

Note: significant (<0.05) *p*-values bolded.

## Data Availability

Underlying data for this study will be made available upon request to the corresponding author (rnataraj@stevens.edu). Data will be provided directly or through a public repository on ResearchGate.
